# Radioactive Iodine Treatment for Thyroid Cancer Complicated by Lacrimal Sac Retention of Iodine

**DOI:** 10.1210/jcemcr/luae234

**Published:** 2024-12-13

**Authors:** Annu Suresh, Giuseppe Esposito, Bruce Davidson, Pavle Doroslovački, Jacqueline Jonklaas

**Affiliations:** Department of Internal Medicine, Medstar Georgetown University Hospital, Washington, DC 20007, USA; Department of Nuclear Medicine, Medstar Georgetown University Hospital, Washington, DC 20007, USA; Department of Otolaryngology-Head and Neck Surgery, Medstar Georgetown University Hospital, Washington, DC 20007, USA; Department of Ophthalmology, Medstar Georgetown University Hospital, Washington, DC 20007, USA; Division of Endocrinology, Medstar Georgetown University Hospital, Washington, DC 20007, USA

**Keywords:** thyroid cancer, radioactive iodine, lacrimal uptake

## Abstract

Patients with intermediate-risk thyroid cancers may undergo treatment with radioactive iodine-131 (I-131). They often undergo a pretreatment diagnostic iodine scan that typically shows areas of physiological uptake in the stomach, bladder, parotid glands as well as thyroid-remnant uptake and sites of metastatic disease. A 48-year-old woman with intermediate-risk papillary thyroid cancer with metastases to lateral compartment lymph nodes was found to have increased retention of iodine in the medial portion of her left orbit on the diagnostic scan. This was suggestive of preexisting nasolacrimal duct stenosis leading to retention of secretions in the lacrimal sac, raising concerns that the I-131 used in treatment would have delayed clearance that could further damage her lacrimal sac and eye. In consultation with ophthalmology, the patient received pretreatment azelastine and prednisolone drops and underwent treatment with radioactive iodine followed by saline lacrimal irrigation. Though she had subsequent eye pain and swelling necessitating repeated irrigation, the patient was able to undergo treatment for her papillary thyroid cancer and retained full function of her eye. This case highlights an approach that could be used for patients with nasolacrimal duct stenosis in whom radioactive iodine treatment is deemed beneficial.

## Introduction

Radioactive iodine is often used in the management of intermediate-risk thyroid cancers. This modality is useful in thyroid-remnant ablation to facilitate further cancer surveillance, adjuvant treatment for destruction of residual subclinical tumor deposits, and treatment of known disease that is not amenable to surgical therapy [1]. Patients who are candidates for treatment with radioactive iodine include patients with extrathyroidal extension, aggressive histological subtypes, or substantial lymph node metastases [1]. Before a patient undergoes treatment with iodine-131 (I-131), some institutional protocols incorporate a pretreatment whole-body scan with either low-dose I-131 or iodine-123 (I-123) to look for areas of metastatic disease or residual cancer burden and assist in selection of radioactive iodine activity.

Some known complications of the treatment include sialadenitis, altered sense of taste, transient decrease in gonadal function, and nasolacrimal duct obstruction. Though less common than other complications, nasolacrimal obstruction can occur with doses as low as 30 mCi (1110 mBq) [2], although the risk increases with higher doses [3]. Subsequently, there can be stenosis of the ducts requiring surgical intervention [2]. The presence of baseline sinonasal disease prior to treatment with I-131 could lead to increased retention and subsequent secretion of radioactive iodine, increasing the risk of acquired obstruction. This case describes a situation in which the pretreatment scan showed increased uptake and retention in the lacrimal sac. The diagnostic scan thus alerted the medical team that the patient was at increased risk for acquired nasal obstruction and possible eye damage with I-131 treatment secondary to radioactivity secreted by lacrimal glands.

## Case Presentation

The patient is a 48-year-old woman with past medical history of Graves disease, polycystic ovary syndrome, and Ehlers-Danlos syndrome who was found to have suspicious thyroid nodules on ultrasound. Family history was notable for a sister with a benign thyroid nodule and father who had hyperparathyroidism now status post parathyroidectomy. Thyrotropin (TSH) was 1.36 μIU/mL (normal, 0.45-4.5 μIU/L) and thyroglobulin was 5.5 ng/mL (normal, 3-40 ng/mL). She had a solid mildly hypoechoic nodule with ill-defined margins and punctate echogenic foci measuring 0.9 × 0.8 × 0.5 cm, fitting a thyroid imaging and reporting data system or TI-RADS score of 5. There was also a cluster of morphologically abnormal lymph nodes with large irregular hila containing punctate echogenic foci similar in appearance to that of the suspicious thyroid nodule at the level of the right lateral level III lymph nodes, the largest of which was 0.9 cm × 0.5 cm. Fine-needle aspiration biopsies both of the nodule and lymph node showed evidence of papillary thyroid cancer (Bethesda Category VI) with metastases to the level III lymph nodes. She underwent thyroidectomy and neck dissection followed by treatment with levothyroxine.

The histology of the 0.5-cm thyroid nodule was papillary carcinoma of the follicular variant with infiltrative features and negative margins. There was no evidence of angioinvasion or extrathyroidal extension. Two of the 42 regional lymph nodes in the right lateral compartment (level III) obtained during the neck dissection were found to be metastatic papillary carcinoma of the thyroid measuring 10 × 5 mm and 1.5 × 1 mm. There was no extranodal extension. Her pathologic stage classification was pT1a and pN1b. Given that her cancer fell within the intermediate-risk category due to infiltrative characteristics and involvement of lateral compartment lymph nodes, her treatment plan also included radioactive iodine.

## Diagnostic Assessment

**Figure 1.**
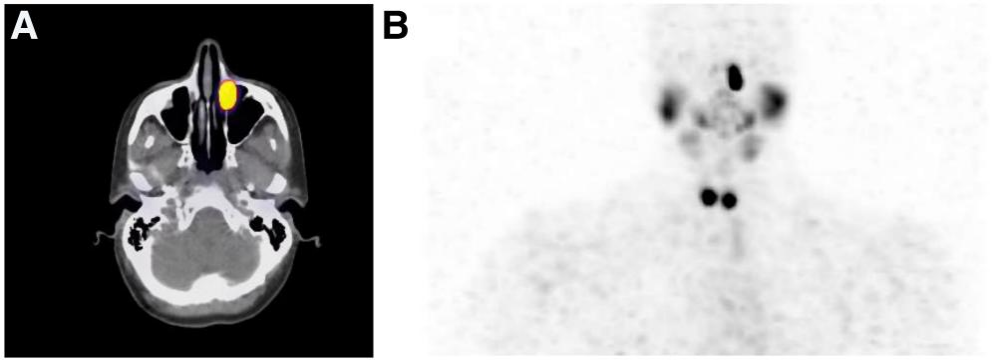
Diagnostic scan with 123-Iodine. A shows the SPECT-CT scan demonstrating increased iodine uptake in the medial portion of the patient's left orbit. B shows the whole-body iodine scan. This scan demonstrates the physiological uptake of the salivary gland and remnant thyroid as well as the irregularity of iodine uptake in the left orbit.

In preparation for treatment with I-131, the patient underwent a recombinant human TSH (Thyrogen)-stimulated diagnostic scan with I-123, during which the stimulated thyroglobulin was 4.7 ng/mL. The pretherapy diagnostic scan (Fig. 1) was notable for a small amount of residual functioning thyroid tissue in the thyroidectomy bed and physiological activity in the salivary glands. Of note, the planar and SPECT-CT images (Fig. 1A and 1B) showed evidence of a large focus of marked radioiodine retention along the inferomedial aspect of the left orbit. On repeat imaging after stimulation of lacrimation, there was only minimal washout of the activity. Following consultation with ophthalmology, it was determined that the patient had a preexisting nasolacrimal duct stenosis, presumptively due to chronic allergic rhinosinusitis, leading to retention of radioactive iodine in her lacrimal duct. While acquired nasolacrimal duct obstruction is a known, though infrequent, complication of I-131 treatment related to the effects of the radioactivity secreted by the lacrimal glands on the lacrimal sac and nasolacrimal duct, the risk of this complication, as well as damage to the ocular adnexa, likely increases with retention of radioactive iodine in the lacrimal sac in the setting of preexisting stenosis.

## Treatment

Given the patient's lateral compartment lymph node involvement and her infiltrative variant of follicular variant of papillary thyroid cancer, it was agreed in discussion between endocrinology, nuclear medicine, surgery, and the patient that she would derive overall benefit from radioactive iodine treatment despite the blockage of the lacrimal sac. The team opted for a slightly higher dose than one purely for remnant ablation given the positive lymph nodes from the neck dissection but did not select a yet higher dose as no further nodal involvement was seen on the diagnostic scan. A preemptive dacryocystorhinostomy was not completed as the risks of this invasive procedure, such as infection, bleeding, chronic dryness, and restenosis, outweighed possible benefits when a less invasive alternative such as eye drops and flushing remained an option. To optimize the patient, she underwent pretreatment with 0.05% azelastine drops in both eyes for 6 weeks up until treatment to reduce allergic inflammation and improved tear drainage. She had a taper of prednisolone 1% drops in the left eye for 1 month to also reduce allergic inflammation.

The patient underwent treatment with an 80 mCi (2960 mBq) dose of I-131 using Thyrogen stimulation. Thirty minutes after treatment, the ophthalmology team performed irrigation of the nasolacrimal duct with normal saline to help facilitate clearance of the radioactive iodine. The logistics of the procedure were coordinated ahead of time. The irrigation process lasted only 4 to 5 minutes and estimated radiation exposure of the operators was well below the regulatory limits.

## Outcome and Follow-up

Three days after treatment, the patient visited the emergency department for worsening left eye pain and new right eye pain with tearing. She was seen by members of the ophthalmology team who attributed this to ocular surface inflammation in response to radioactive iodine. She underwent bilateral nasolacrimal duct irrigation that was notable for rapid efflux on the right indicating a patent nasolacrimal duct and moderate reflux with delayed saline clearance on the left indicating persistent stenosis. She started a posttreatment regimen of 0.05% azelastine drops, artificial tears, and a 1-week taper of prednisolone 1%.

Although the patient's symptoms improved, she developed recurrent left eye pain associated with eye swelling and blurry vision 4 days later. This was again thought to be due to the effects of radioactivity on the ocular surface. She again required nasolacrimal duct irrigation. Beyond these acute symptoms within the first 2 weeks post treatment, the patient has not had any further ocular complications in the last 10 months. She has been undergoing posttreatment surveillance with thyroglobulin measurements and serial neck ultrasounds without evidence of recurrence.

## Discussion

While sialadenitis, xerostomia, transient amenorrhea, and increased risk of secondary malignancies are all well-known complications of radioactive iodine treatment, nasolacrimal duct obstruction and associated problems are less commonly considered. Prior studies have suggested a rate of approximately 2.2% patients treated with radioactive iodine who develop acquired nasolacrimal duct obstruction [4]. Use of artificial tears after radioactive iodine treatment has been suggested as a strategy to decrease the level of radioactivity [5]. Guidelines suggest referral to ophthalmology for symptoms of xerophthalmia or epiphora, but do not address prevention [6]. Utilization of the lowest dose of radioactive iodine expected to be effective appears to be a wise approach, as lower doses generally have fewer side effects [2, 3].

Though no prior studies have assessed the rate of this complication in patients with preexisting lacrimal duct stenosis, it is anticipated that the risk would be increased. On one hand, a patient who reports issues with recurrent eye infections, tearing, and crusting may have a baseline degree of duct stenosis or propensity for this complication. However, there are likely to be other patients who present without these preexisting clinical features but have evidence of iodine retention on imaging. While patients diagnosed with nasolacrimal duct stenosis can be treated surgically, no clear protocols have been established to prevent nasolacrimal duct obstruction in patients at higher risk. Prevention using topical saline drops and nasolacrimal duct massage have been considered, but the efficacy of these interventions has not been assessed [4]. S-2-(3-243 aminopropylamino)-ethylphosphorothioic acid (amifostine) has been reported to reduce salivary gland damage after radioactive iodine treatment but has not been assessed as a means of preventing damage [7]. Rather than delaying radioiodine therapy in patients with sinonasal conditions or patients found to have preexisting stenosis, the intervention used for this patient offers an immediate and easily coordinated solution. Future patients with preexisting nasolacrimal stenosis could undergo a similar protocol with pretreatment and posttreatment eye drops with peritreatment lacrimal duct irrigation.

This case also highlights a benefit in utilization of pretreatment diagnostic scanning with radioactive iodine. A diagnostic scan is part of our institutional protocol as in certain cases it alters therapy including deciding against radioactive iodine treatment based on minimal uptake, increasing or titrating the therapeutic dose based on unexpected uptake in lymph nodes or distant metastases, and delaying therapy or use of dopamine agonistics in patients with breast uptake after pregnancy or lactation [8]. Our case highlights an added benefit of the pretreatment scan: the ability to identify possible complications and trial preventive measures.

Regarding limitations, one cannot fully confirm that the treatment regimen used for this patient had an effect on the risk of complications associated with radioactive iodine, given that it was used on only one patient without a comparator. Another limitation of applying the experience from our case report is that we could not compare different regimens to know if another regimen may have provided better results. Also, the regimen used for this patient did not entirely prevent short-term side effects. Beyond this, there is no guarantee that the immediate irrigation prevented long-term effects of the retention or radiation, but hopefully some insight will be gained during this patient's follow up.

## Learning Points

- Lacrimal glands are a site of physiological uptake of iodine.
- Nasolacrimal duct obstruction and dry eye, rare complications of high-dose I-131 radioactive iodine treatment, are more likely to occur in patients with preexisting nasolacrimal duct stenosis as the lacrimal sac will retain and secrete radioactivity in tears.
- Repeated nasolacrimal irrigation following treatment with I-131 in a patient with nasolacrimal duct stenosis may be an effective way to prevent further obstruction or ocular damage in a patient for whom radioactive iodine treatment is indicated.

## Contributors

All authors made individual contributions to authorship. A.S. wrote the first version of the manuscript with subsequent input from all other authors. J.J., G.E., B.D., and P.D. were involved in the diagnosis and management of the patient. B.D. was responsible for the patient's thyroid surgeries. G.E. was responsible for the nuclear portions of the patient's diagnosis and treatment. P.D. provided ophthalmologic care. All authors reviewed and approved the final draft of the manuscript.

## Data Availability

Data sharing is not applicable to this article as no data sets were generated or analyzed during this study.

## Abbreviations

I-123, iodine-123; I-131: iodine-131
TSH: thyrotropin
